# Enhancing oxygen evolution efficiency of multiferroic oxides by spintronic and ferroelectric polarization regulation

**DOI:** 10.1038/s41467-019-09191-0

**Published:** 2019-03-29

**Authors:** Xiaoning Li, Huan Liu, Zezhi Chen, Qingmei Wu, Zheyin Yu, Mengmeng Yang, Xiaolin Wang, Zhenxiang Cheng, Zhengping Fu, Yalin Lu

**Affiliations:** 1 National Synchrotron Radiation Laboratory, University of Science and Technology of China, 230026 Hefei, China; 2 Institute for Superconducting & Electronic Materials (ISEM), Australia Institute for Innovative Materials, Innovation Campus, University of Wollongong, Squires Way, North Wollongong, NSW 2500 Australia; 3 Chinese Academy of Sciences (CAS) Key Laboratory of Materials for Energy Conversion, Department of Materials Science and Engineering, University of Science and Technology of China, 230026 Hefei, China; 4 Synergetic Innovation Center of Quantum Information and Quantum Physics & Hefei National Laboratory for Physical Sciences at Microscale, University of Science and Technology of China, 230026 Hefei, China

## Abstract

Regulating the electronic structure of catalysts is the most efficient strategy yet, despite its limitations, to improve their oxygen evolution efficiency. Instead of only adjusting the electronic structure, here we utilize ferroelectric polarization to accelerate the oxygen evolution reaction as well. This is demonstrated on a multiferroic layered perovskite Bi_5_CoTi_3_O_15_ with in-situ grown BiCoO_3_. Thanks to the superimposed effects of electronic regulation and ferroelectric polarization, the as-prepared multiferroic electrocatalysts are more efficient than the benchmark IrO_2_ (with a final 320 mV overpotential at the current density of 10 mA cm^−2^ and a 34 mV dec^−1^ Tafel slope). This work not only demonstrates a low-cost and high-efficient OER electrocatalyst, but also provides a strategic design for multi-component electrocatalytic material systems by consideration of both spin and polarization degrees of freedom.

## Abstract (web summary)

While splitting water into fuel may provide a green, renewable method for energy storage, water oxidation is its bottleneck. Here, authors reported multiferroic electrocatalysts with improved oxygen evolution performances assisted by polarization.

## Introduction

The oxygen evolution reaction (OER) process not only requires four sequential proton-coupled electron transfers, but also includes a transition of the spin states from the singlet OH^−^/H_2_O to the triplet O_2_^1,2^. Such a spin transition needs to be promoted by an additional energy to occur, enough voltage together with spin orbital coupling for example. So, OER has been the rate-determining step that hinders applications in various energy conversion and storage devices^3^. Considering the scarcity and high cost of commercial OER catalysts, such as Pt and magnetic RuO_2_/IrO_2_, a great deal of effort has been expended on possible alternatives^4^. Transition metal-based perovskite is one of the most excellent candidates, owing to its abundance, relatively good stability, and benign electrocatalytic activity^5^. In 2011, a volcano-shaped curve of OER efficiency against the number of *e*_g_ electrons of surface transition metal cations has been proposed by Yang Shao-Horn et al^6^. Following this rule, the fastest OER is observed on Ba_0.5_Sr_0.5_Co_0.8_Fe_0.2_O_3–δ_ (BSCF), of which the magnetic ions are in the intermediate spin state, that is, the number of *e*_g_ electrons is around 1.2. Since then, this principle has become an effective guideline to improve the efficiency of perovskite oxide OER catalysts^5^. For example, Zeng’s group successfully adjusted the nanoparticle size of LaCoO_3_ to be around 80 nm and the *e*_g_ number is manipulated to 1.2, resulting in the reduced overpotential (330 mV) and Tafel slope (69 mV dec^–1^)^7^. Wu’s groups adjusted the *e*_g_ electron number of LaCoO_3_ films to 1.2 by  introducing different degrees of octahedral distortion, resulting in an overpotential of 470 mV^8^. Yang Shao-Horn’s group also acquired insight into several different coordination configurations based on transition metal oxides, such as a distorted prism, corner sharing, and face-sharing. However, whether this rule is applicable in the composites remains unclear., since  composites with a combination of two or more different phases may provide flexible tuning of average *e*_g_ electron numbers in contrast to the rigid single phase.

Meanwhile, more strategies should also be explored to further improve the efficiency considering the limitations of *e*_g_ electron number regulation. Ultrathin two-dimensional (2D) morphology engineering for maximum surface area is one of the most effective approaches that have been recently developed^9^. For example, a cobalt oxyhydroxide nanosheet with an atomic thickness performs 20 times higher than that of its bulk counterpart and also exceeds the benchmark IrO_2_ electrocatalyst^10^. Even so, it must be pointed out that an atomic two-dimensional morphology usually requires subtle and intricate multistep soft chemistry routes with low production. Excitingly, it has been discovered that the internal polarization of ferroelectrics can be taken advantage of to separate the carriers^11–16^. Meanwhile, ferroelectrics can also produce an external screening effect, adsorbing charged ions and molecules from outside to neutralize their polarization inside^17–21^, which may provide a valuable way to enhance the adsorption capacity. Unfortunately, ferroelectric polarization is not yet fully utilized in the OER process. Inspired by the above-mentioned spin state effects and the ferroelectric polarization function, we considered the multiferroic layered perovskite oxides as an excellent material matrix to combine the two strategies together to further improve the OER efficiency^22,23^. Being perovskite derivatives, layered perovskites preserve multiple perovskite physical parameters, such as couplings between charge, spin, orbitals, and the lattice, and have attracted tremendous attention for multiferroic and energy conversion devices^24,25^. More importantly, layered perovskite oxides have higher degrees of freedom in their structure and are amenable to modulation of properties by component substitution or layer number regulation.

Herein, we propose a method to improve the OER activity: in situ growth of a secondary phase on a ferroelectric matrix to tune the *e*_g_ electron number and utilizing the ferroelectric polarization by corona poling afterward. The material system is designed from the deliberate insertion of several layers of BiCoO_3_ (BCO) into the three-layered perovskite Bi_4_Ti_3_O_12_ (BTO). From considerations of structural tolerance and thermodynamic stability, only one layer of BCO can be inserted to form a four-layered perovskite oxide Bi_5_CoTi_3_O_15_ (BCTO), while the residual BCO would be deposited in situ on its surface as the secondary phase. This configuration provides a suitable research platform to reveal the significance of the electronic regulation, as well as ferroelectric polarization on the OER performance. It turned out to be very effective, resulting in a superior OER performance with 34 mV dec^–1^ Tafel slope and 320-mV overpotential to deliver a current density of 10 mA cm^–2^. The electronic structure and the contribution of the ferroelectric polarization are studied in detail by various tests and measurements, and a possible enhanced mechanism is proposed.

## Results

### Structure and morphology

**Fig. 1:**
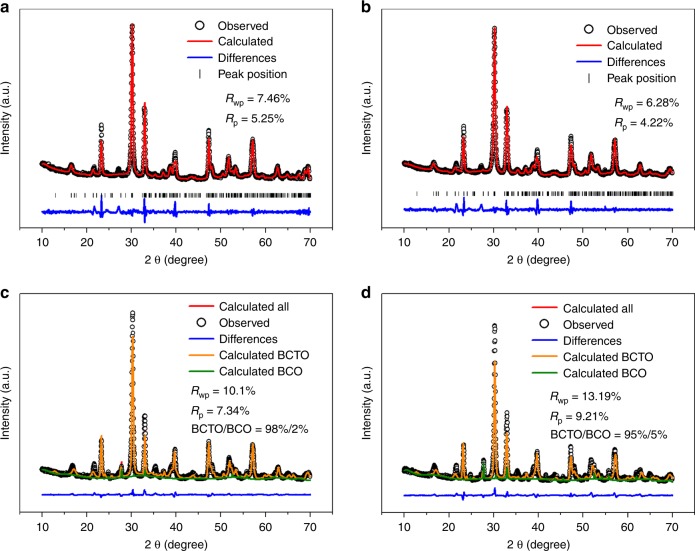
XRD patterns and refinements for as-prepared samples. **a** Co1; **b** Co2; **c** Co3; and **d** Co4

Four samples were prepared by the in situ hydrothermal method according to the designed composition of Bi_4_Ti_3_O_12_·(BiCoO_3_)_*n*_ (*n* = 1, 2, 3, 4), and denoted as Co1, Co2, Co3, and Co4, respectively. The morphologies are displayed in Supplementary Figure 1. From the SEM images, we can see that the as-prepared samples are mainly composed of 10-nm-thick nanoplates (with the average width varying from 1 to 2 μm), yet with tiny nanoparticles on the surface. The quantity of nanoparticles on the surface increases from Co1 to Co4. XRD patterns were collected to identify their compositions and are displayed in Fig. 1. The refinements of the Co1 and Co2 samples were analyzed by the Pawley method based on the four-layer perovskite BCTO crystal structure (tetragonal, space group F2mm (42), JCPDS 38–1257). The resultant profile R-factor (*R*_p_) values are far smaller than 10%, indicating that Co1 and Co2 samples are primarily BCTO. As shown in Supplementary Figure 2c, this crystal structure is an alternation of four layers of perovskite-like oxide with one layer of fluorite-like bismuth oxide. In octahedral coordination with O, Co, and Ti cations can occupy the A sites randomly in the perovskite-like sublattice.

According to our previous work, collapse of a perovskite-like sublattice will inevitably occur when too many perovskite-like cobalt oxide layers are inserted, resulting in a decreased number of layers and the appearance of a secondary phase. Although the amount of the secondary phase in the Co1 and Co2 samples is too negligible to quantify by XRD refinement, we can discriminate the secondary phase by the appearance of a peak at 27.8°. This peak can be indexed to BiCoO_3_ or some other isostructures, such as Co-deficient Bi_24.96_CoO_40_ (in all BiCoO_3_ isostructures, Co and Bi ions are tetrahedrally coordinated, and if there are more Bi ions, they will occupy the Co sites, or vice versa, as shown in Supplementary Figure 2a, b). So, the refinements of the Co3 and Co4 samples are analyzed by the Pawley method based on two phases: BCTO and BCO. From the refinement results, there are 2% BCO in the Co3 and 5% BCO in the Co4 sample. All lattice constants are listed in Supplementary Table 1. Thus, the results from XRD suggest that when one or two BiCoO_3_ layers are inserted into a three-layered perovskite Bi_4_Ti_3_O_12_, the structure will turn out to be a four-layered perovskite BCTO; but when three or four layers are inserted, a 2% or 5% secondary phase BCO will appear.

**Fig. 2:**
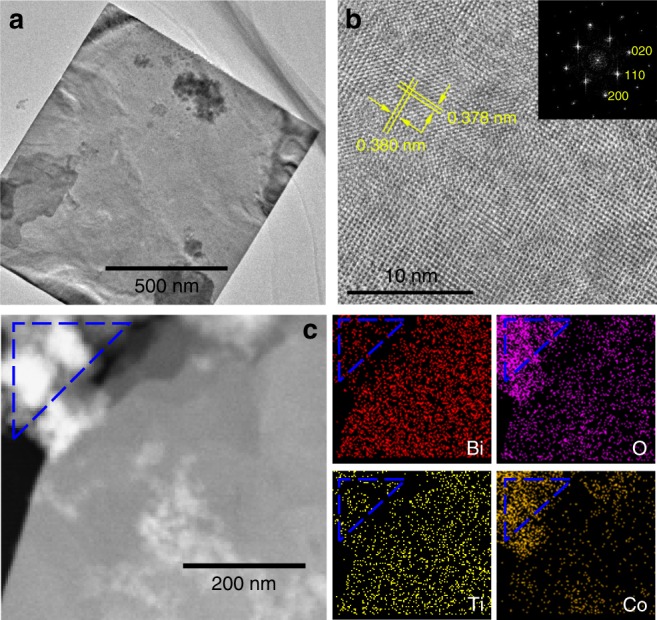
Morphology of the Co2 sample. **a** TEM image to show the typical morphology; **b** HRTEM image of the nanoplate in Fig. 2a with the inset of the corresponding SAED pattern; **c** STEM image and the corresponding EDS element mapping images

The typical TEM image in Fig. 2a collected from the Co2 sample shows the morphology. Figure 2b is a high-resolution TEM (HRTEM) image of a BCTO nanoplate recorded with the electron beam along the [001] zone axis. The spacings of the observed lattice are about 0.378 and 0.380 nm, consistent with the (110) crystalline planes of the orthorhombic four-layer perovskite phase. The selected area electron diffraction (SAED) pattern obtained from the corresponding area in the inset of Fig. 2b shows sharp diffraction spots, indicating that the BCTO nanoplates are single crystals with good crystallinity. Figure 2c contains a high-angle annular dark-field scanning transmission electron microscope (HAADF) image together with EDS mapping to investigate the distribution of elements for the main phase and the secondary phase. Apparently, Bi, Ti, Co, and O are distributed homogeneously throughout the BCTO nanoplates as well as the BCO nanoparticles, but there are more Bi, Co, and O signal dots in the nanoparticles than in the nanoplates, indicating that the secondary phase nanoparticles are indeed Bi–Co-enriched BCO, justifying the results of the XRD patterns.

The above results mean that we have successfully prepared four samples composited by the in situ grown secondary phase BCO nanoparticles and the four-layer structured BCTO nanoplates. The BCO ratio increases gradually for composites Co1, Co2, Co3, and Co4, ranging in 0−5 wt% of the total mass of each sample. In order to better show the advantages of the in situ as well as to  identify role of each phase on the following OER tests, we also synthesized the pure Bi_4_Ti_3_O_12_ (BTO), pure BCO, and pure BCTO samples for comparsion. Ex situ sample was prepared  by physically mixing the pure BCO and pure BCTO with a ratio of 2.5 wt%, and denoted as Phy Mix (Supplementary Methods, XRD and SEM, see Supplementary Figure 3 and Supplementary Figure 4).

### Electrocatalytic water splitting

**Fig. 3:**
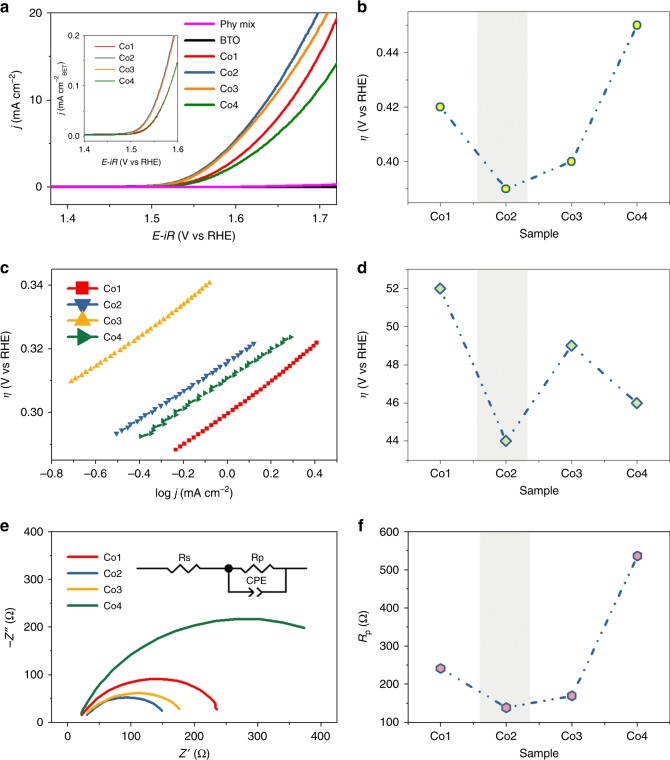
Electrocatalytic characterization for the OER. **a** LSV curves obtained at the scan rate of 5 mV s^-1^; the inset is the corresponding normalized curve based on the BET surface area; **b** overpotential of the four samples at *η*_*j*=10_; **c** Tafel plots; **d** Tafel slopes of the four samples; **e** Nyquist plots, with the inset showing the equivalent circuit; and **f** polarization resistance (*R*_p_) of the four samples

The OER activities of the four in situ grown samples (Co1, Co2, Co3, and Co4), Phy Mix sample, and the reference BTO sample were characterized by linear-scan voltammetry (LSV) experiments in 1 M NaOH electrolyte (Fig. 3a). The current density at 1.7 V vs. RHE is 20.34 mA cm^–2^, 0.25 mA cm^–2^, and 0.025 mA cm^–2^ for Co2, Phy Mix, and the BTO sample, respectively. Obviously, the performances of the in situ grown samples are several orders higher than those of the physically mixed sample and the reference BTO sample. This can be explained by the following reasons. At first, in situ grown secondary phase nanoparticles on the surface of nanoplates ensure a favorable heterojunction interface, which renders a fast electron transfer as well as a possible synergistic effect of the two phases. Moreover, the unique morphology, that is, decoration of the nanoparticles of BCO on the nanoplate of BCTO, guarantees abundant active sites (BET specific surface areas are 22.2 m^2^ g^–1^, 25.53 m^2^ g^–1^, 22.79 m^2^ g^–1^, and 17.56 m^2^ g^–1^ for Co1, Co2, Co3, and Co4, respectively), which is another reason for the incredible enhancements of in situ samples compared with those of ex situ sample.

It is worth noting that the Co2 and Co3 samples exhibit similar current density (*j*) after the normalization based on the BET surface areas (inset of Fig. 3a), which will be discussed in detail with more characterizations. The potential at current density *j* = 10 mA cm^–2^ (*η*_*j*=10_) is one of the main indicators characterizing the efficiency of a cell. The corresponding overpotentials (against 1.23 V vs. RHE) of the in situ grown samples are plotted in Fig. 3b, which decreased from 420 mV to 387 mV, and then increased up to 452 mV. Since a lower overpotential means less energy consumed to split the water, the Co2 sample with the lowest overpotential (387 mV at *j* = 10 mA cm^–2^) is the highest-performing OER electrocatalyst of all. The Tafel plots in Fig. 3c and the Tafel slope values in Fig. 3d further confirm that the Co2 sample possesses the smallest Tafel slope of 44 mV dec^–1^. Such small Tafel slopes (≤52 mV dec^–1^) indicate that the rate-determining step tends to change from –OH adsorption to –OOH formation^6^, according to the possible OER process (Equation 1–4).

1 $${\mathrm{Catalyst}} + 2{\mathrm{OH}}^ - = {\mathrm{Catalyst}} - {\mathrm{O}} + {\mathrm{H}}_2{\mathrm{O}} + 2e^ -$$

2 $${\mathrm{Catalyst}} - {\mathrm{O}} + {\mathrm{OH}}^ - = {\mathrm{Catalyst}} - {\mathrm{OOH}} + e^ -$$

3 $${\mathrm{Catalyst}} - {\mathrm{OOH}} + {\mathrm{OH}}^ - = {\mathrm{Catalyst}} - {\mathrm{OO}} + {\mathrm{H}}_2{\mathrm{O}} + e^ -$$

4 $${\mathrm{Catalyst}} - {\mathrm{OO}} + {\mathrm{OH}}^ - = {\mathrm{Catalyst}} - {\mathrm{OH}} + {\mathrm{O}}_2 + e^ -$$

The measured electrochemical impedance spectra (EIS) provide more information about the electrochemical process. Nyquist plots can be resolved by an equivalent circuit, which is composed of *R*_s_, *R*_p_, and CPE, as shown in Fig. 3e. Here, *R*_s_ is the solution resistance, *R*_p_ is the polarization resistance, and CPE represents a constant phase element. The Co2 sample has the smallest polarization resistance and the *R*_p_ values of the four samples are shown in Fig. 3f, which is in line with the results from LSV. In addition, the electrochemical active surface area (ECSA) is positively related with a double-layer capacitance *C*_dl_, which can be calculated based on the Nyquist plot parameters and the equation

5 $$C_{{\mathrm{dl}}} = (\frac{T}{{R{\mathrm{s}}^{P - 1}}})^{\frac{1}{P}}/S$$

*T*, *P*, and *R*_s_ are the parameters resolved from Nyquist plots, and the details are provided in Supplementary Table 2. The calculated *C*_dl_ is ~82 μF cm^–2^, 158 μF cm^–2^, 107 μF cm^–2^, and 124 μF cm^–2^ for the Co1, Co2, Co3, and Co4 samples, respectively. Although these four samples have a similar morphology and BET specific surface areas, the Co2 sample possesses a larger ECSA (*C*_dl_) than that of the other three samples.

### Electronic structure analysis

**Fig. 4:**
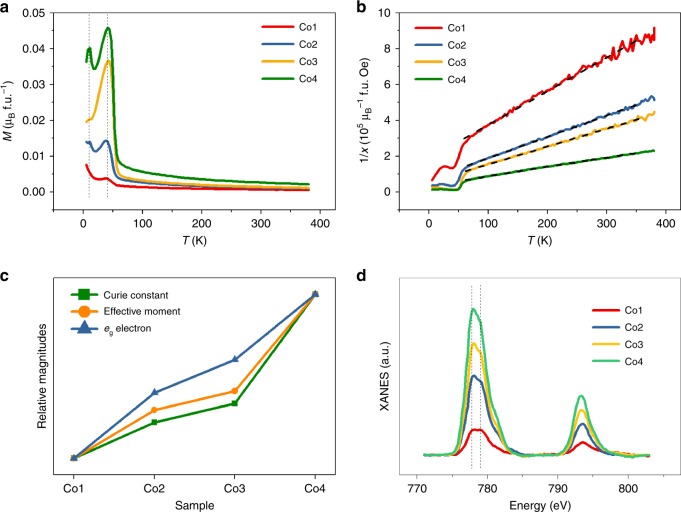
Electronic structure analysis. **a** Temperature dependence of the magnetization curves (*M*−*T*); **b** inverse susceptibility 1/*χ* against temperature; **c** tendency of *C*, *µ*_eff_, and *e*_g_ electron number among the four samples; **d** normalized Co *L-*edge XAS spectra

The O 1*s* spectra of all samples were shown in Supplementary Figure 5, which includes two peaks located at 529.6 and 531.4 eV. Lattice oxygen contributes to the relatively higher peak absorption (529.6 eV), while the latter shoulder peak should stem from the less electron-rich oxygen species, probably the surface chemisorbed oxygen species on the surface of BCO and BCTO^26^ (e.g., O−, surface OH groups, contamination of CO_2_, etc.). The peak area ratio of surface/lattice oxygen was defined as *R*_so_, and it is about 50%, 59%, 56%, and 55% for the Co1 to Co4 samples. The relatively high percentage of surface-adsorbed oxygen species indicates that all the samples should have a strong chemisorbing capacity. In addition, the Co2 sample exhibited the best adsorption capacity, which is likely to have originated from its superior surface electronic structure, which will be discussed later. To provide further information, the temperature dependence of the magnetization (*M*−*T*) was measured under a magnetic field of 500 Oe. There are two broadened peaks located at 10 and 42 K in Fig. 4a, which are caused by weak antimagnetic interactions of Co−O−Co. The Curie−Weiss law (Eq. 6) was applied to fit the curves, as shown in Fig. 4b. The effective magnetic moment *µ*_eff_ can be calculated according to Equation 7, while the average *e*_g_ electron number can be derived by Equation 8 from the *J* value with the assumed quenching of orbital angular momentum (*L* = 0) and a measured *g* factor^27^.

6 $$\frac{1}{\chi } = \frac{1}{C}T + \frac{{T_C}}{C}$$

where *χ* refers to the magnetic susceptibility, *C* is Curie−Weiss constant (*C* > 0), *T* is temperature, and *T*_C_ is the Curie point.

7 $$\mu _{{\mathrm{eff}}} = \sqrt {\frac{{3kC}}{N}}$$

where *µ*_eff_, *k*, *C*, and *N* are the effective magnetic moment, Boltzmann constant, Curie−Weiss constant, and the number of magnetic ions in the unit cell, respectively.

8 $$\mu _{{\mathrm{eff}}} = g\sqrt {J\left( {J + 1} \right)} \mu _{\mathrm{B}}$$

where *g* is the Lande factor (regarded as 4.2 in Bi_6_Fe_2_Ti_3_O_18_ and Bi_6_FeCrTi_3_O_18_^28^), the total quantum number *J* equals to the spin quantum number *S* when *L* = 0, and *μ*_B_ means the Bohr magneton.

Figure 4c demonstrates the increasing trends in *C*, *μ*_eff_, and *e*_g_ number from the Co1 to the Co4 samples (for the numerical values, see Supplementary Table 3). In theory, to reveal the evolution of the electronic structure, the secondary phase BCO and the main phase BCTO should be separated when measuring, which was very difficult to realize in our case, however. Nevertheless, the ratio of the BCO to BCTO is relatively very small (0−5 wt%), and the only difference between the four samples is none other than the ratio of BCO to BCTO. Thus, it is reasonable to identify the contribution of the secondary phase BCO by carefully comparing the subtle change in the entire BCO/BCTO sample. Here, the increase in the calculated average *e*_g_ electron number from the Co1 to the Co4 sample is due to the increased of amount of BCO. In BCO, the Co ions are tetrahedrally coordinated with threefold degenerate high-energy (HE) *t*_2g_ orbitals and low-energy (LE) doubly degenerate *e*_g_ orbitals. In BCTO, the Co ions are octahedrally coordinated with doubly degenerate high-energy *e*_g_ orbitals and threefold degenerate low-energy *t*_2g_ orbitals. Therefore, the calculated *e*_g_ number actually means the average electron number in high-energy orbitals (HE), that is,  the mixture of  *e*_g_ from the main phase BCTO and *t*_2g_ from 0 to 5 wt% BCO.

XAS is an effective technique to investigate the specific electronic occupation. As shown in Fig. 4d, the similar peaks featuring in the Co *L*_2,3_ edge spectra means that all the Co is in the same valence state. Owing to the spin–orbit splitting, Co 2p state can bring about two absorption peaks (778 and 779 eV). Even though most octahedrally coordinated transition metal ions are suggested to be in the low-spin state (LS) due to the strong crystal field energy, the octahedrally coordinated Co^3+^ ions (O_Co_) from the BCTO nanoplates have a strong probability of being found in the intermediate spin (IS) state with the configuration of (*t*_2g_)^5^(*e*_g_)^1^, since they would make no contribution to the absorption peak at 778 eV if they were in the LS state (*t*_2g_)^6^(*e*_g_)^0^. In the tetrahedral coordination of the secondary phase BCO, Co^3+^ ions (T_Co_) may be in the high-spin (HS) state (*e*_g_)^3^(*t*_2g_)^3^ on account of the low crystal field of the tetrahedron. Then, the absorption peak at 778 eV is relatively influnced by the LE orbital, that is, the 2*p*−*t*_2g_ transition of HS T_Co_. Meanwhile, the absorption peak of 779 eV is more related to the HE orbital, that is, the 2*p*−*e*_g_ transition of IS O_Co_. In this situation, the relative intensity of the peak at 778 eV will be increased if the ratio of BCO to BCTO is increased, just as the results of XAS measurements suggest. Since there are 3 electrons in the HE orbital of the BCO while there are only 1 electron in the HE orbital of the BCTO, the average HE electron number (*e*_g_ number calculated above) will be increased with the increase of BCO,  in agreement with the results based on the *M*−*T* curves.

### Influence of ferroelectric polarization

**Fig. 5:**
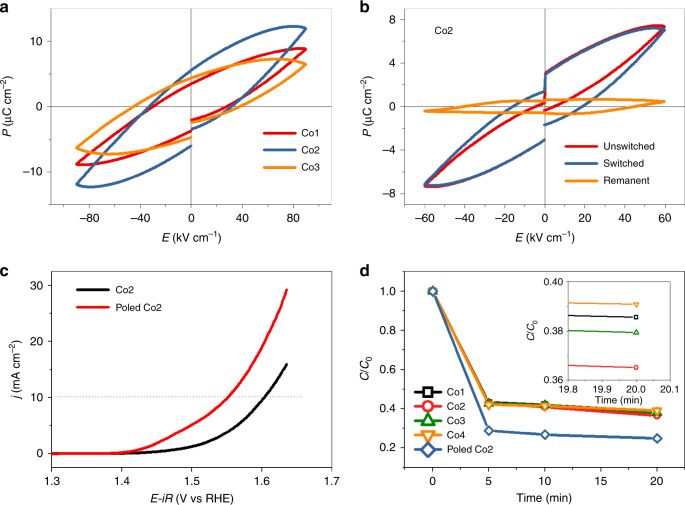
Effects of ferroelectric polarization. **a** *P*–*E* loops at the maximum electric field of 90 kV cm^−1^ measured at room temperature; **b** remnant *P*–*E* loop of Co2 at the maximum electric field of 60 kV cm^−1^ measured at room temperature; **c** LSV curves of Co2 after corona poling; **d** adsorption curves of the RhB dye for the four samples as well as the poled Co2 sample, with the inset of the enlarged residual concentration at the adsorption equilibrium after 20 min

Figure 5a presents the polarization−field (*P*−*E*) loops at room temperature. The measured *P*−*E* loops are unable to reach saturation and they are not closed, all due to a certain degree of current leakage. In fact, leakage problem is very common for smaller bandgap ferroelectric materials (especially the Bi–Co-contained oxides). Despite the leakage, the intrinsic ferroelectric polarization of a layered perovskite material family has been well recognized^29^. The saturation polarization (2*P*_s_) is 24.5 μC cm^–2^ for the Co2 sample at the applied field of 80 kV cm^–1^. The remnant polarization (2*P*_r_) extracted from the *P*−*E* loops in Fig. 5a increases from 7.12 to 11.59 μC cm^–2^ and then decreases to 9.11 μC cm^–2^  from Co1, Co2 to Co3, indicating the best ferroelectricity of Co2 sample among others . Supplementary Figure 6 contains the hysteresis *P*−*E* loops with increasing poling electric fields, from which it is found that the nominal remnant polarization is enhanced correspondingly. This suggests that the domains in the samples have the potential to be activated and oriented by applying a strong enough electric field. Figure 5b is the remnant *P*−*E* loop of the Co2 sample (the corresponding remnant *P*−*E* loops for the other three samples are shown in Supplementary Figure 7). Compared with the standard *P*−*E* measurement, remnant *P*−*E* loops will be more reliable and provide more information about the reverse voltage, reverse speed, and useful non-residual polarization. The red curve represents the unswitched polarization part, the blue curve the switched part, and the yellow one the remnant part (the difference between the switched and unswitched polarizations). The closed feature of the remnant *P*−*E* loop further confirms the benign ferroelectric properties of the Co2 sample.

As the layered perovskite nanoplates are single crystalline and ferroelectric, they will easily exhibit a single-domain structure aligned along the poling electric field direction. The corona polar method was applied to form the one-directional dipolar alignment inside (for the self-made setup, see Supplementary Figure 8). Figure 5c presents the LSV curves measured on the Co2 sample before and after poling, respectively. Remarkably, the overpotential at 10 mA cm^–2^ is reduced from 387 mV to 320 mV after poling. The superimposed improvements by poling can be explained as follows. At first, the poled crystals should attract more electrically charged species from the ambient environment on their surfaces to screen the enhanced internal built-in electric field for the sake of charge neutrality. Therefore, a larger remnant polarization may result in better adsorption capability. To test this inference, the adsorption capacity of the four samples was measured with the organic dye rhodamine B (RhB) as the adsorbate target. As displayed in Fig. 5d, all the as-prepared ferroelectric samples can adsorb about 60% of the RhB molecules in 10 mg L^–1^ RhB solution, and the Co2 sample shows superior adsorption over the other three (inset of Fig. 5d), with another 20% improvement after poling. In addition, the energy band of the poled single crystals will inevitably be bent by the magnitude of 0.3−0.5 V, as estimated in BaTiO_3_ at room temperature^15^. The OER process might be accelerated at the same applied voltage with a favorable energy band bending.

**Fig. 6:**
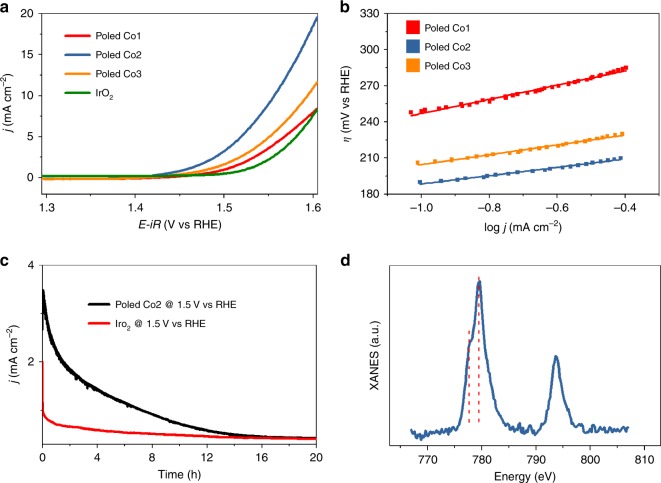
Electrocatalytic characterization for the corona-poled samples. **a** LSV curves at the scan rate of 5 mV s^−1^; **b** Tafel plots; **c** long-term chronoamperometric measurement of poled Co2 and IrO_2_; **d** Co *L*-edge XAS spectra of the poled Co2 sample before chronoamperometric measurement

To verify the above assumption, Co1 and Co3 samples were also corona poled and their LSV curves and Tafel plots were shown in Fig. 6a and 6b. Based on the LSV curves in Fig. 6a, the overpotential at the current density *j* = 10 mA cm^–2^ (*η*_*j*=10_) is about 390 mV, 320 mV, and 360 mV for Co1, Co2, and Co3, respectively. Apparently, poled Co2 and Co3 have exceeded the state-of-the-art commercial IrO_2_, which is with 380 mV *η*_*j*=10_. Corona poling treatment reduced the η_j=10_ values by 30 mV, 70 mV, and 40 mV for the Co1, Co2, and Co3, respectively. Moreover, the Tafel slope is decreased to be 59 mV dec^–1^, 34 mV dec^–1^, and 40 mV dec^–1^ for the three poled samples (Fig. 6b). ECSA is calculated in the same method as mentioned above and the details are provided in Supplementary Table 2, from which we can see that ECSA does not change after corona poling treatment. LSV curves normalized by the BET surface area as well as the ECSA are presented in Supplementary Figure 9, and their tendency is consistent with the ferroelectric polarization results, indicating that the differences in the OER performance between the three poled samples are mainly originated from the intrinsic polarization intensity.

As shown in Supplementary Figure 10, 20 cycles of cyclic voltammetry (CV) were conducted on the poled Co2 sample, and there is no obvious redox peak during the forward and backward sweep, suggesting a very stable phase and structure. However, the performance of the 20th cycle is relatively worse than that of the 1st cycle. A long-term chronoamperometric measurement was also conducted on the poled Co2 sample at the potential of 1.5 V vs. RHE with the duration of 20 h (Fig. 6c). The measured corresponding current density decreased gradually in the first 14 h and stabilized afterward. After careful analysis, we found that the initial current density (3.5 mA cm^–2^) is almost the same as that from the LSV curve for the poled Co2 sample in Fig. 6a (3.4 mA cm^–2^ at 1.5 V vs. RHE). But 14 h later, the current density is decreased to 0.45 mA cm^–2^ (eightfold decreased). To find the underlying mechanism, TEM and HRTEM images of the poled Co2 sample before and after the 20-h chronoamperometric measurement were conducted (Supplementary Figure 11). TEM images demonstrate that the poled Co2 sample still keeps its original morphology during the 20-h test. HRTEM images confirm its regular lattice fringe and crystalline surface morphology, and SAED patterns verify that there is no amorphization or phase transformation to other compositions, such as CoOOH and so on. All these results suggest that there is no morphology or phase structure change during either the corona poling or the long-term OER test.

Then the XAS spectrum of the corona poling-treated poled Co2 sample before the chronoamperometric measurement was measured and presented in Fig. 6d to gain in-depth surface electronic information. It is obvious that the peak intensity at 779 eV is much higher than that of 778 eV in the poled Co2 sample before the long-term chronoamperometric measurement, which is in sharp contrast to the curves in Fig. 4d. According to the above analysis, it means that the concentration of HE  Co increased when poling the Co2 sample. This change could be due to a possible surface electronic structure modification during the corona poling process before the long-term test. The discharged air plasma in the corona poling process may ensue a tangling bond, defect generation, charge injection, and other modifications of the surface, accompanied by the ferroelectric poling. After the 14-h long-time chronoamperometric measurement, this kind of transient surface electronic structure modification might be decreased. However, the poled Co2 sample still performed better than the unpoled Co2 sample and IrO_2_ with the assistance of a permanent ferroelectric polarization inside in this situation (0.45 mA cm^–2^ of poled Co2, 0.17 mA cm^–2^ of poled Co2, and 0.32 mA cm^–2^ of IrO_2_). It means that the performance improvement contributed by the surface electronic modification may be dwindled gradually within 14 h, but the effects contributed by the ferroelectric polarization work all the time.

## Discussion

**Fig. 7:**
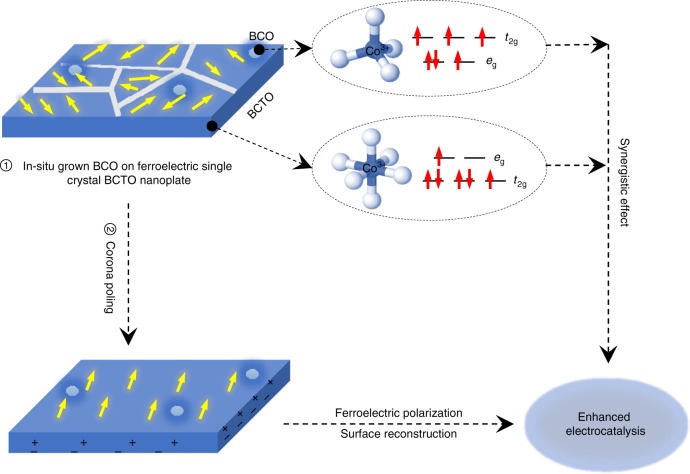
Illustration to demonstrate the advantages of the in situ as-prepared BCO/BCTO nanostructures for electrocatalysis. The Co ions of the in situ grown BCO are tetrahedrally coordinated with (*e*_g_)^3^(*t*_2g_)^3^, while Co ions of the ferroelectric BCTO are octahedrally coordinated with (*t*_2g_)^5^(*e*_g_)^1^, and these two different configurations have a synergistic effect on the overall OER efficiency. By corona poling, the ferroelectric polarization together with an induced surface modification can greatly enhance the OER performance

The high intrinsic OER efficiency and similarity of the LSV curves after normalization by BET surface area for Co2 and Co3 can be attributed to the compromises of ferroelectric polarization and spin states. The Co2 sample is with better ferroelectricity, while the effective magnetic moment *μ*_eff_ (the average *e*_g_ electron number) of the Co3 sample is higher. But after poling, Co2 performed better than Co3 as the contribution of the ferroelectric polarization is dominant in this situation. Figure 7 presents the idea of this work: by inserting several magnetic layers of BCO into a ferroelectric layered perovskite, 0−5 wt% BCO-decorated four-layered perovskite BCTO nanoplates were formed. The Co^3+^ in the BCO is in the tetrahedrally coordinated (*e*_g_)^3^(*t*_2g_)^3^ HS state, which increases the *e*_g_ electron number (or high-energy orbital electrons). Meanwhile, BCTO ultrathin nanoplates provide a large surface area and Co^3+^ in the IS spin state with the (*t*_2g_)^5^(*e*_g_)^1^ configuration, as well as a desirable remnant polarization. During the OER process, it is the electrons located in the unfulfilled high-energy orbitals of the 3*d* transition metal ion on the surface that participate in σ-bonding with the adsorbates. Neither too weak nor too strong bonding is favorable for absorption and desorption at the same time. Due to its intrinsic ferroelectric property, BCTO nanoplates may present an approximate single-domain structure with a strong polarity when applying the corona poling. This provides additional driving forces toward charge transport on the electrode due to band bending and attracts more charged interactants, such as OH^−^ near the surface in the electrocatalytic OER process. At the same time, corona poling might also bring about a favorable surface modification, which further improves the OER efficiency of the electrocatalysts.

 In summary, we reported a strategy to improve the OER activity: the in situ growth of a secondary phase on a ferroelectric matrix and corona poling it afterward. At first, layered perovskite BCTO nanoplates with in situ grown BCO nanoparticles were successfully prepared by a one-step hydrothermal method. According to the XRD and SEM results, the BCTO nanoplates are 10 nm in thickness, and the BCO nanoparticles are in the ratio of 0−5 wt%. HRTEM and EDS mapping confirm the single crystallinity of the BCTO nanoplates and the composition of the BCO nanoparticles. All four samples exhibit excellent electrocatalytic OER activity, low overpotential, low Tafel slopes, and low polarization resistance. Nevertheless, the efficiency is increased from the Co1 to the Co2 sample, and then decreased on the Co3 and Co4 samples, although they have a similar BET surface area (about 20 m^2^ g^–1^). Based on the *M*−*T* and XAS results, the average *e*_g_ electron number is increased from the Co1 to the Co4 sample. The maximum OER activity was realized on the Co2 sample with its appropriate electronic configuration. What is more interesting, the OER property can be further improved by the corona poling method, resulting in an overpotential of 320 mV to deliver a 10 mA cm^–2^ current density and a Tafel slope of 34 mV dec^–1^. The phase and morphology are very stable after treatment by corona poling and the long-term OER tests. The improvement contributed by the surface modification may be decreased gradually within 14 h, but the effects of ferroelectric polarization work all the time. This work may provide an additional instructive strategy to enhance the OER performance for future energy conversion and storage devices.

## Methods

### Synthesis

Samples in the present work were prepared by a simple hydrothermal method. A certain amount of Ti(OC_4_H_9_)_4_ (>99.7%) was dissolved into 4 M HNO_3_ solution (7.5 mL) forming a uniform solution (around 1.2 g), and then the amount of other raw materials (Bi(NO_3_)_3_·5H_2_O (>99%) and Co(NO_3_)_2_·6H_2_O (>99%)) should be calculated based on the stoichiometric ratio according to Bi_4_Ti_3_O_12_·*n*(BiCoO_3_) (*n* = 1, 2, 3, 4), with the products denoted as Co1, Co2, Co3, and Co4, respectively, and then added into the above solution. The homogeneous metal-ion solution was magnetically stirred for 20 min, and was added into a 70-mL NaOH solution with a concentration of 1.66 M. After another 20 min of stirring, the resulting suspension was transferred into a Teflon-lined stainless-steel autoclave (total capacity of 100 mL). Then the autoclave was sealed and heated at 200 °C for 2 days. The sediment was washed with water and ethanol several times and then dried at 60 °C for 8 h.

### Material characterization

X-ray powder diffraction patterns (XRD) were recorded on the Rigaku-TTR III X-ray diffractometer with Cu-Kα radiation. Scanning electron microscopy (SEM, JSM-6700F) and transmission electron microscopy (TEM, JEM-2010), and an aberration-corrected scanning transmission electron microscope (STEM) (JEM-ARM200F, JEOL) equipped with X-ray energy-dispersive spectroscopy (EDS) (X-max80, Oxford Instruments) were applied to identify the morphologies. The Brunauer–Emmett–Teller (BET) surface area was estimated by using the adsorption data (Tristar II 3020 M, Mircomeritics, USA). The magnetic properties were measured on a Quantum Design physical property measurement system (PPMS) (Quantum Design, USA) with the vibrating sample magnetometer (VSM) option. The electronic structure was analyzed based on the X-ray photoelectron spectroscopy (XPS) from the ESCALAB 250 system (Thermo Scientific), and soft X-ray absorption spectra (XAS) from BL12B-a facility of the National Synchrotron Radiation Laboratory (NSRL, P. R. China). The ferroelectric properties (*P*−*E* loops) were tested on a Precision LC ferroelectric analyzer (Radiant Technology Product, USA). The corona poling method was applied on the Co2 sample. The voltage of the steel-point electrode was 22 kV, and the 0.01-g Co2 powder was uniformly coated on a negative disk-like copper electrode with an area of about 12 cm^2^. The distance between the two electrodes is 2 cm. Since the poled area at any one time was about 2 cm^2^, the poling experiment is conducted six times on different parts of the disk-like copper electrode coated with the Co2 sample, and the duration was 2 min.

### Electrochemical testing

The OER performance was obtained on an electrochemical workstation (CHI instruments 660E, China) with a standard three-electrode electrochemical cell. The saturated Ag/AgCl was used as the reference electrode, a platinum wire as the counter electrode, and a glassy carbon electrode coated with the electrocatalyst is the working electrode. The working electrode was prepared according to the following steps: (1) mixing 0.75 mL of deionized water and 0.25 mL of methanol; (2) adding 10 mg of an electrocatalyst and 0.1 mL of 5 wt% Nafion^®^ solution into the above solution; (3) dispersing it in the ultrasound for 1 h to form a mixed ink; (4) drop-casting 3 μL of the above suspension into a glassy carbon electrode that is 3 mm in diameter (effective area 7.065 mm^2^); and (5) drying naturally. The final working electrode is with a loading of 0.38 mg cm^–2^.

LSV curves, CV curves, and Tafel plots were obtained at a scan rate of 5 mV s^–1^. Long-term chronoamperometric measurements were conducted at 1.5 V vs. RHE with a scan rate of 10 mV s^–1^. In order to measure the Tafel plot, an open potential (*V*_o_) was measured at first. Then Tafel plots were obtained under a Tafel mode and the range was set to *V*_o_~*V*_o_ + 100 mV with a scan rate of 5 mV s^–1^. Electrochemical impedance spectroscopy (EIS) was conducted with the condition of 1.686 V vs. RHE, which is 10^6^ Hz to 0.1 Hz.

All the potentials vs. Ag/AgCl were converted into RHE according to the Nernst Equation 8:

8 $$E_{{\mathrm{RHE}}} = E_{{\mathrm{Ag}}/{\mathrm{AgCl}}} + E_{{\mathrm{Ag}}/{\mathrm{AgCl}}}^0 + 0.059 \ast {\mathrm{pH}}$$

where *E*_RHE_ refers to the potential vs. RHE, $E_{{\mathrm{Ag}}/{\mathrm{AgCl}}}^0$ is 0.1976 V, and *E*_Ag/AgCl_ is the applied potential vs. Ag/AgCl. In the present work, *E*_RHE_ are corrected by subtracting *iR* (where *i* is the current, and *R* equals to the *R*_s_ value resolved from Nyquist plots).

## Supplementary information

Supplementary Information

## Data Availability

The data that support the findings of this study are available from the corresponding author upon request.
