# Supplementary material for: Enhancing oxygen evolution efficiency of multiferroic oxides by spintronic and ferroelectric polarization regulation
Source: Nat Commun. 2019 Mar 29;10:1409. doi: 10.1038/s41467-019-09191-0 (PMC6441026; doi:10.1038/s41467-019-09191-0)
Supplement: Supplementary file 1 — Supplementary Information [file 41467_2019_9191_MOESM1_ESM.pdf]

Supplementary Information

Enhancing oxygen evolution efficiency of multiferroic oxides by spintronic and ferroelectric polarization regulation

By Li *et al*

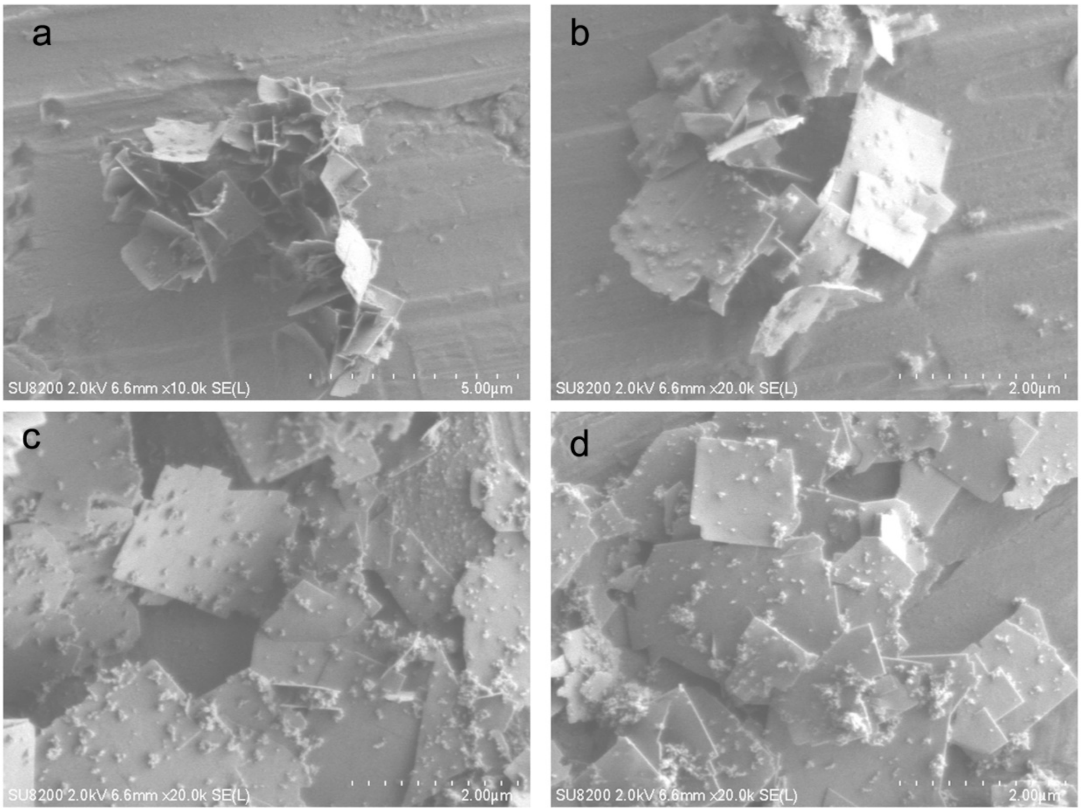

Supplementary Figure 1 | SEM images of as-prepared samples. a Co1; **b** Co2; **c** Co3 and **d** Co4.

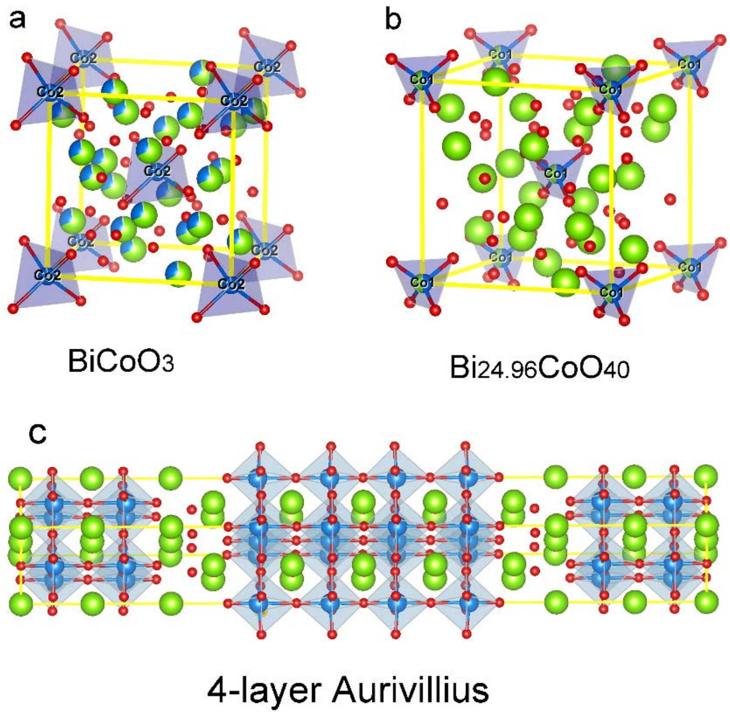

Supplementary Figure 2 | Crystal structures of $BiCoO_3$, $Bi_{24.96}CoO_{40}$, and $Bi_5CoTi_3O_{15}$. Red spheres present O, green spheres present Bi, and blue spheres present 3d transition metal Co (or Co/Ti in Aurivillius structure)

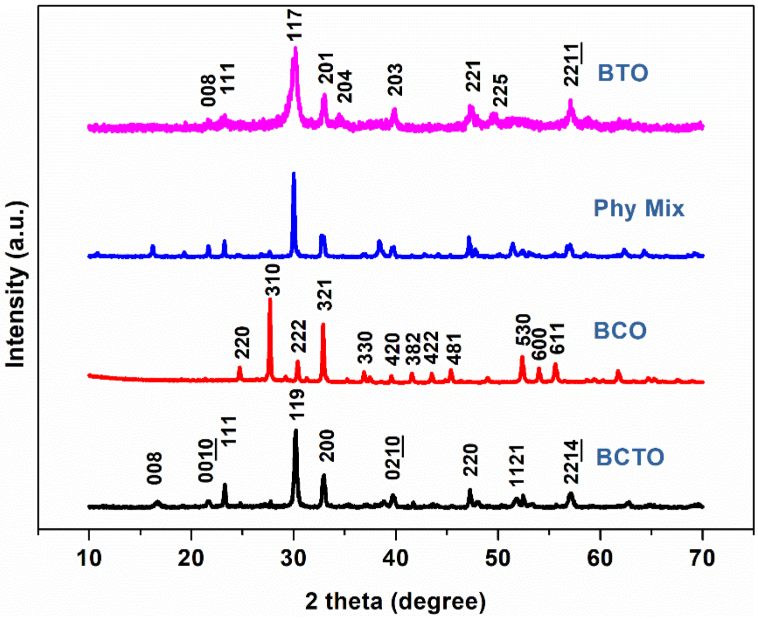

Supplementary Figure 3 | XRD patterns of pure BCTO, BCO, Phy Mix (physically mixed 2.5 wt% BCO and BCTO), and BTO samples.

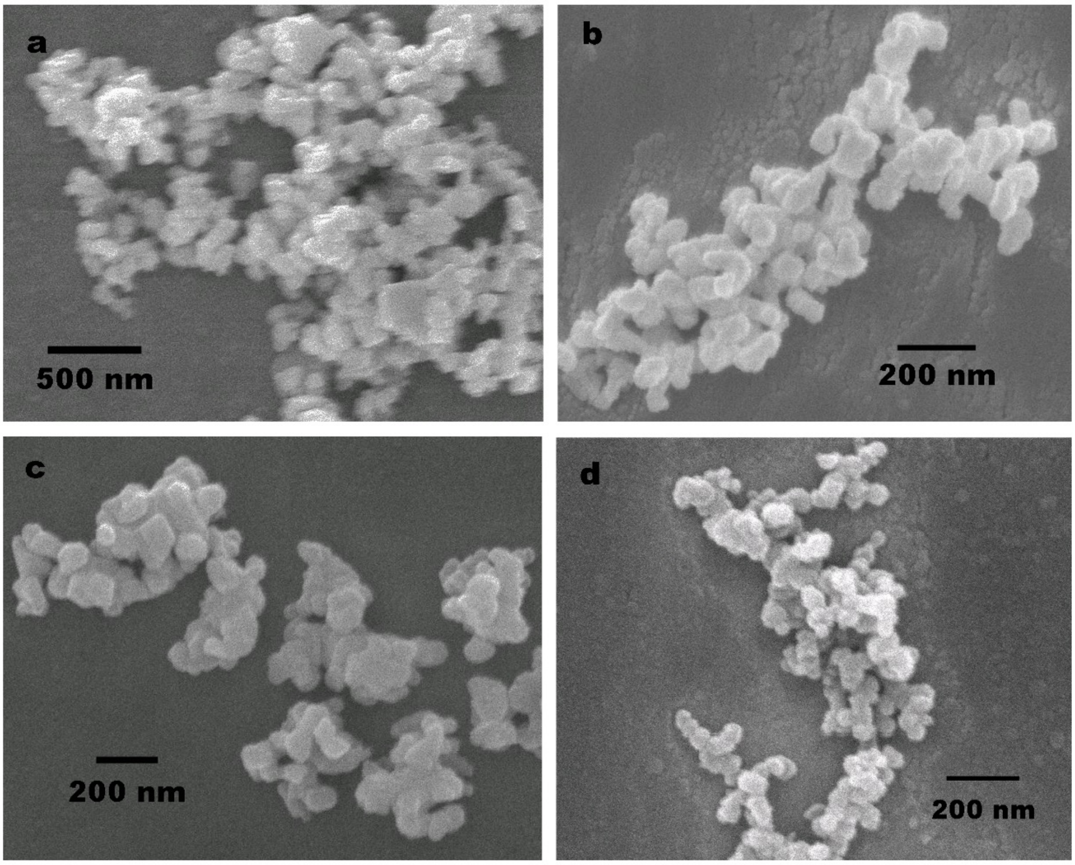

Supplementary Figure 4 | SEM images. a pure BCTO; **b** pure BCO; **c** Phy Mix (physical mixed 2.5 wt% BCO and BCTO), and **d** pure BTO samples.

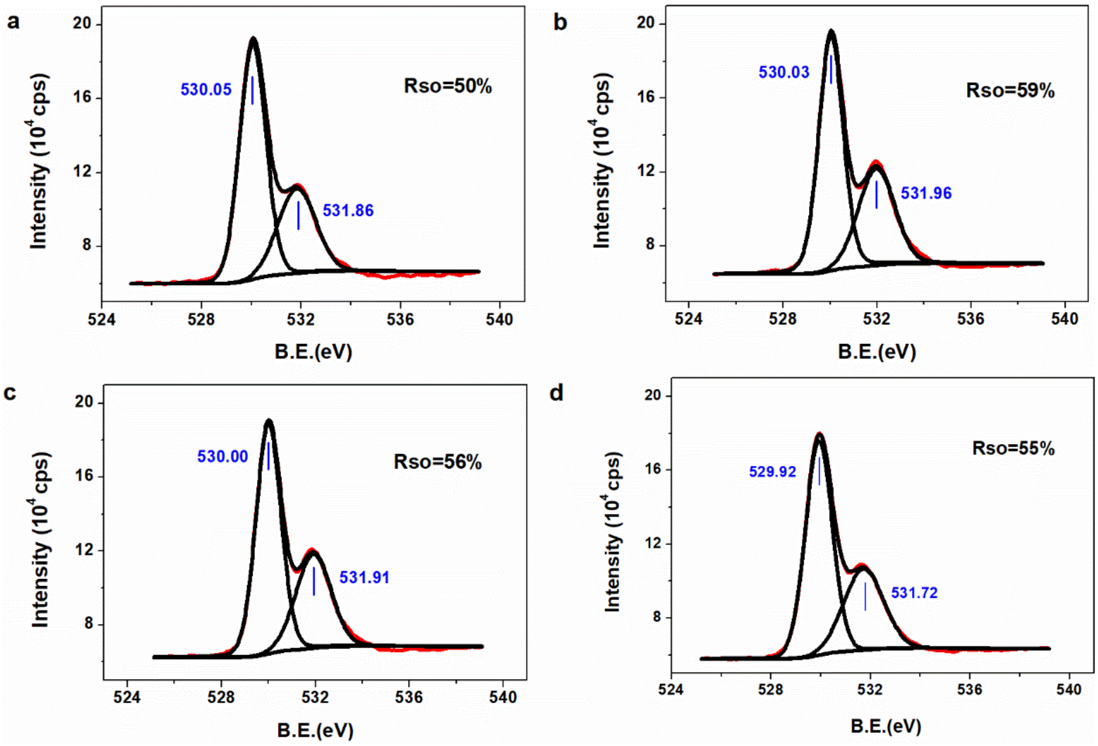

Supplementary Figure 5 | XPS spectra of O 2*p* peak. a Co1; **b** Co2; **c** Co3 and **d** Co4

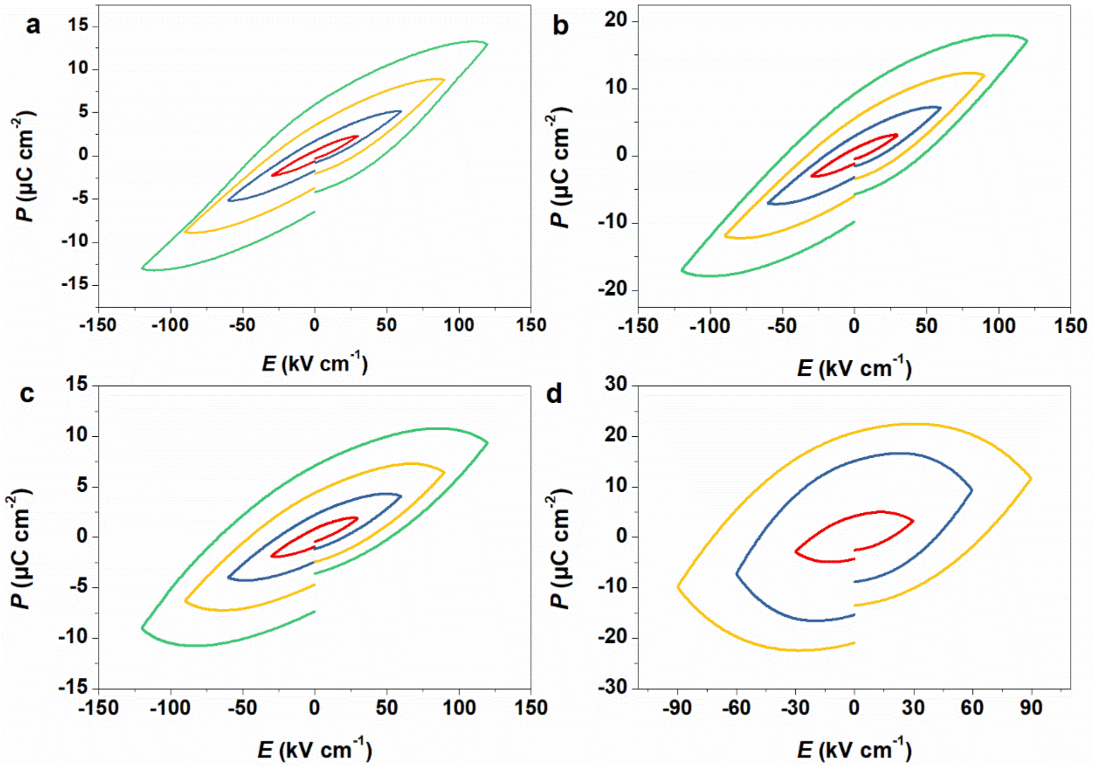

Supplementary Figure 6 | *P*–*E* loops under different applied electric fields at room temperature. a Co1; **b** Co2; **c** Co3 and **d** Co4.

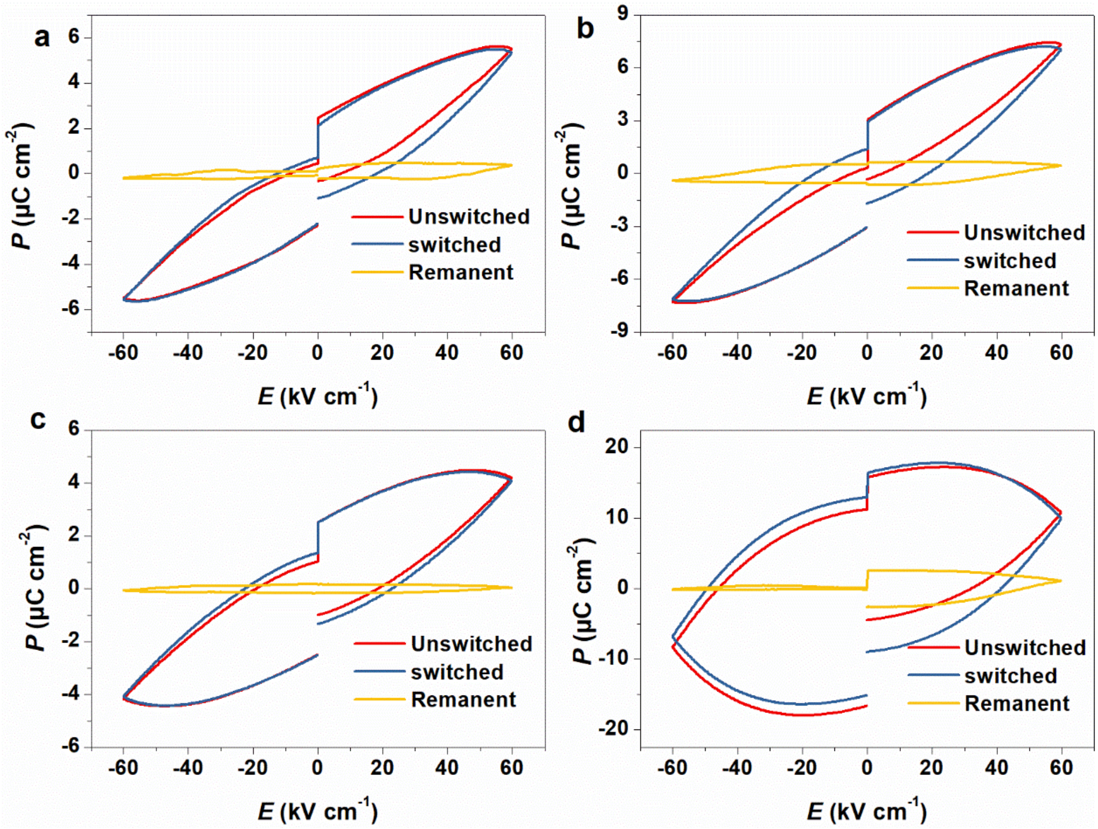

Supplementary Figure 7 | Remnant *P*–*E* loops at 40 kV cm^{-1} at room temperature. a Co1; **b** Co2; **c** Co3 and **d** Co4.

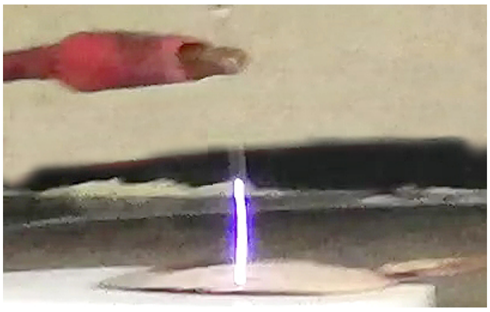

Supplementary Figure 8 | Self-made setup for corona poling. The applied voltage is 22 kV, the distance between two electrodes is about 2 cm.

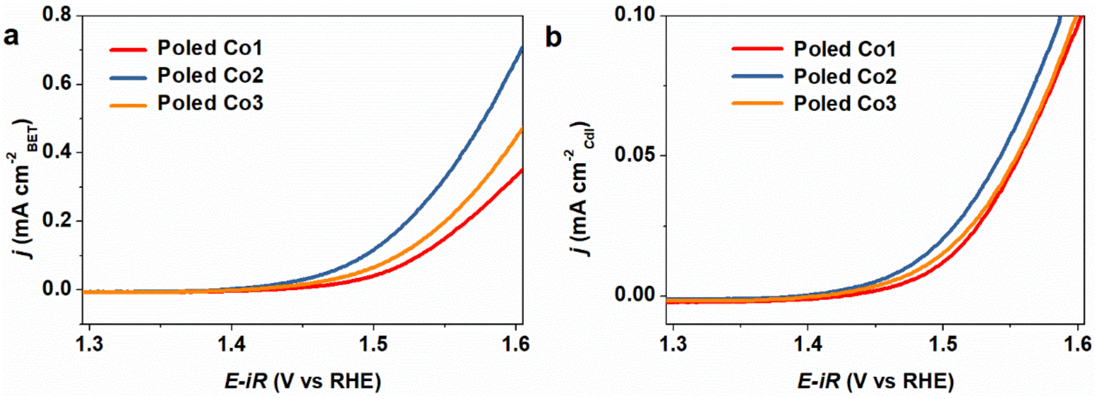

Supplementary Figure 9 | Normalized LSV curves. a by BET surface area of the poled Co1, Co2 and Co3 samples; **b** by ECSA (C_{dl}) of the poled Co1, Co2 and Co3 samples.

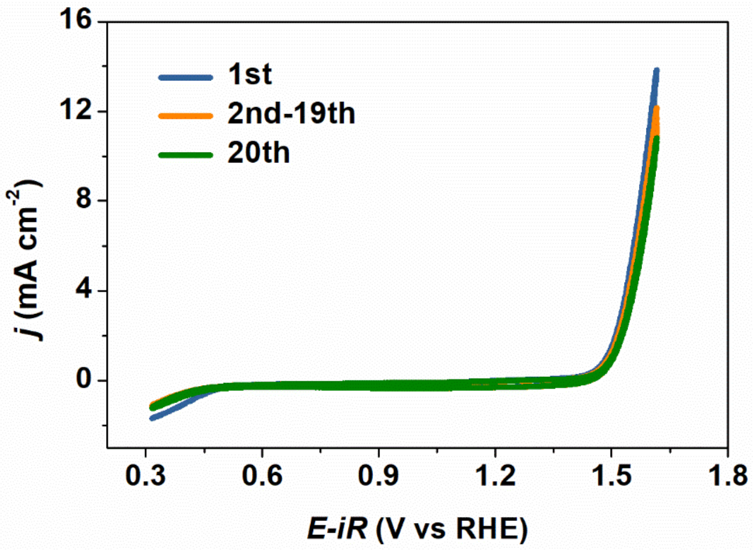

Supplementary Figure 10 | 20 cycles of cyclic voltammetry (CV) on the poled Co2 sample.

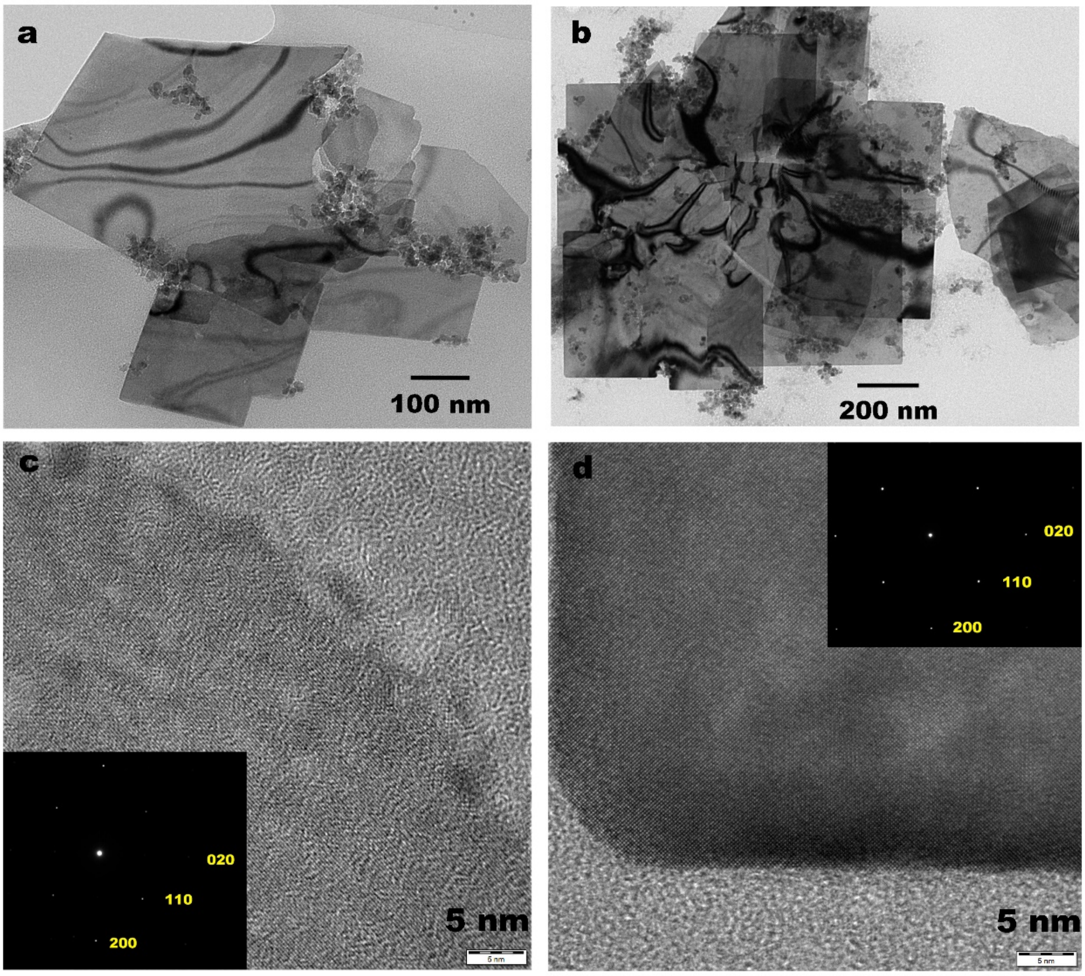

Supplementary Figure 11 | Morphology before and after 20 hours chronoamperometric measurement. a TEM image of poled Co2 sample before chronoamperometric measurement; **b** TEM image of poled Co2 sample after chronoamperometric measurement; **c** HRTEM image and corresponding SAED of the main phase BCTO of the poled Co2 sample before chronoamperometric measurement; **d** HRTEM image and corresponding SAED of the main phase BCTO of the poled Co2 sample after chronoamperometric measurement.

Supplementary Table 1 | Cell constants of the main phase BCTO refined by Pawley method.

**Sample**	***a*** **(Å)**	***b*** **(Å)**	***c*****(Å)**
**Co1**	5.383	5.351	41.11
**Co2**	5.430	5.412	41.55
**Co3**	5.431	5.436	41.56
**Co4**	5.411	5.426	41.25

Supplementary Table 2 | Parameters by analyze Nyquist plots to calculate the capacity C_{dl}.

Sample	*Rs* (Ω)	(CPE) *T*	(CPE) *P*	C_{dl} (μF cm^{-2})
**Co1**	17.43	3.0E-5	0.82	82
**Co2**	24.36	4.7E-5	0.82	158
**Co3**	22.6	4.0E-5	0.81	107
**Co4**	17.07	3.0E-5	0.86	124
**Poled Co1**	23.55	3.7E-5	0.79	79
**Poled Co2**	28.56	3.0E-5	0.87	143
**Poled Co3**	38.32	3.7E-5	0.81	106

Here, C_{dl} is calculated based the Supplementary Equation 1:

$$C_{dl} = \left(\frac{T}{Rs^{P-1}}\right)^{\frac{1}{P}}/S \quad (1)$$

Where S is the area of electrode, equals 0.07065 cm^2.

For the two parameters T and P, they are based on Supplementary Equation 2. T, the frequency independent parameter and P ($0 < P < 1$), the deviation from the ideal behavior, being P= 1 for perfect capacitors and P= 0 for pure resistance.

$$Z_{CPE} = \frac{1}{T \times jw^{P}} \qquad (2)$$

Supplementary Table 3 | Estimated Curie-Weiss constant *C*, effective magnetic moment μ_{eff} and e_g electron number.

Sample	C (10^{-4} K μ_B Oe^{-1} $f.u.^{-1}$)	μ_{eff} (μ_B)	e_g electron
**Co1**	5.32	4.65	1
**Co2**	8.40	5.85	1.2
**Co3**	10.02	6.32	1.3
**Co4**	19.38	8.73	1.5

Supplementary Methods

Preparation of the BCO, BCTO, Phy Mix, and BTO.

In a typical synthesis, $Ti(OC_4H_9)_4$ (> 99.7%), $Bi(NO_3)_3 \cdot 5H_2O$ (> 99%), $Co(NO_3)_2 \cdot 6H_2O$ (> 99%) were dissolved into 4 M HNO_3 solution according to the stoichiometric ratio of $Bi_4Ti_3O_{12}$, $BiCoO_3$, and $Bi_5CoTi_3O_{15}$, respectively. After 20 minutes of magnetic stirring, the homogeneous metal-ion solution was added into a 1.66 M NaOH solution (70 mL). The resulting slurry was aged overnight and then washed with water and ethanol several times. Afterwards, it was dried at 60 °C for 8 hours and sintered at 650°C for 2 hours. According to the stoichiometric ratio, samples are denoted as BTO, BCO and BCTO. Phy Mix was prepared by physically mixing BCO and BCTO powders with the ratio of 2.5 wt% using an agate mortar.
